# Holmium Yttrium-Aluminum-Garnet Laser Lithotripsy: An Effective Endoscopic Treatment for Bouveret’s Syndrome

**DOI:** 10.5152/tjg.2024.23552

**Published:** 2024-03-01

**Authors:** Hasan Selim Güler, Oğuz Üsküdar

**Affiliations:** Department of Gastroenterology, Çukurova University School of Medicine, Balcalı Hospital, Adana, Turkey

Dear Editor,

A 72-year-old woman was consulted at the emergency department with malaise, severe nausea, persistent episodes of vomiting, and loss of appetite for a week. On physical examination, she had epigastric tenderness, and her vital values were stable except for mild tachycardia (112 beats/min). Her medical history included hypertension and no previous surgery. An abdominal computed tomography scan was performed, which revealed dilated stomach, pneumobilia, gallbladder wall thickening, and an impacted mass in the duodenum (see Figures 1 and 2). Upper endoscopy was subsequently performed. Distal esophagus was circumferentially eroded, the stomach was filled with fluid and mixed food. There was a large bile stone in the bulb of duodenum and it was impassable into the second part of duodenum (see Figure 3). An attempt was made to retrieve the stone endoscopically using a basket; however, removal was unsuccessful. As a result, alternative treatment options were discussed with the patient, leading to the decision to proceed with endoscopic laser lithotripsy. Prior to the procedure, written informed consent was obtained. Consequently, a second endoscopic procedure was performed, utilizing holmium laser lithotripsy. A 550-µm holmium laser probe (Boston Scientific) was employed that continuously used in urological interventions. The holmium laser fiber was passed through the working channel of the gastroscope using a cannula designed for an endoscopic retrograde cholangiopancreatography instrument. The laser parameters were configured to a pulse energy of 2 J, a power output of 30 W, and a frequency of 15 Hz. The single session procedure lasted for 4 hours, and the stone was fragmented and taken out piecemeal (see Figure 3). Subsequently, the scope advanced into the second section of duodenum (see Figure 4). All symptoms were resolved following the procedure without complication.

Bouveret’s syndrome is a specific condition of gastric outlet obstruction due to giant gallstone. The first case report was in 1896 by Leon Bouveret. It is very rare approximately 0.5% and is generally reported in elderly females.^1^ Symptoms are nonspecific, and physical findings are often subtle. Computed tomography is generally more useful for diagnosis; diagnosis can also be made by direct radiography and upper endoscopy. Laboratory tests are not helpful for this diagnosis. Various surgical and endoscopic procedures are available for the treatment. Endoscopic approach is rarely used but is more recommended in order to avoid surgery which is accompanied by high mortality and morbidity rates.^2^ Bouveret’s syndrome is typically noted in elderly female patients with concurrent comorbid conditions. Consequently, surgical intervention carries a heightened risk of mortality and morbidity. Endoscopic baskets and/or some miscellaneous lithotripsy techniques (mechanical, laser, electrohydraulic (EH), and extracorporeal shockwave (ESW)) are useful in Bouveret’s syndrome. The unaccompanied endoscopic basket is ineffective in removing large stones, often requiring adjunct techniques such as lithotripsy.^2^ For large, impassable stones, the surrounding tissue may be damaged during mechanical, EH, and ESW lithotripsy. In contrast, endoscopists can target precisely the stone with minimal tissue damage when using laser lithotripsy.^3^ Various lasers, such as holmium:yttrium aluminum garnet (YAG), rhodamine, and neodymium are employed, particularly in the treatment of urinary stones, while data regarding their application for gallstones remain limited. Among these, the holmium: YAG laser stands out as more effective, being the most commonly utilized and researched in this context.^4^ In conclusion, if available, laser lithotripsy can be priority recommended as an effective, promising therapeutic treatment for Bouveret’s syndrome.

## Figures and Tables

**Figure 1.**
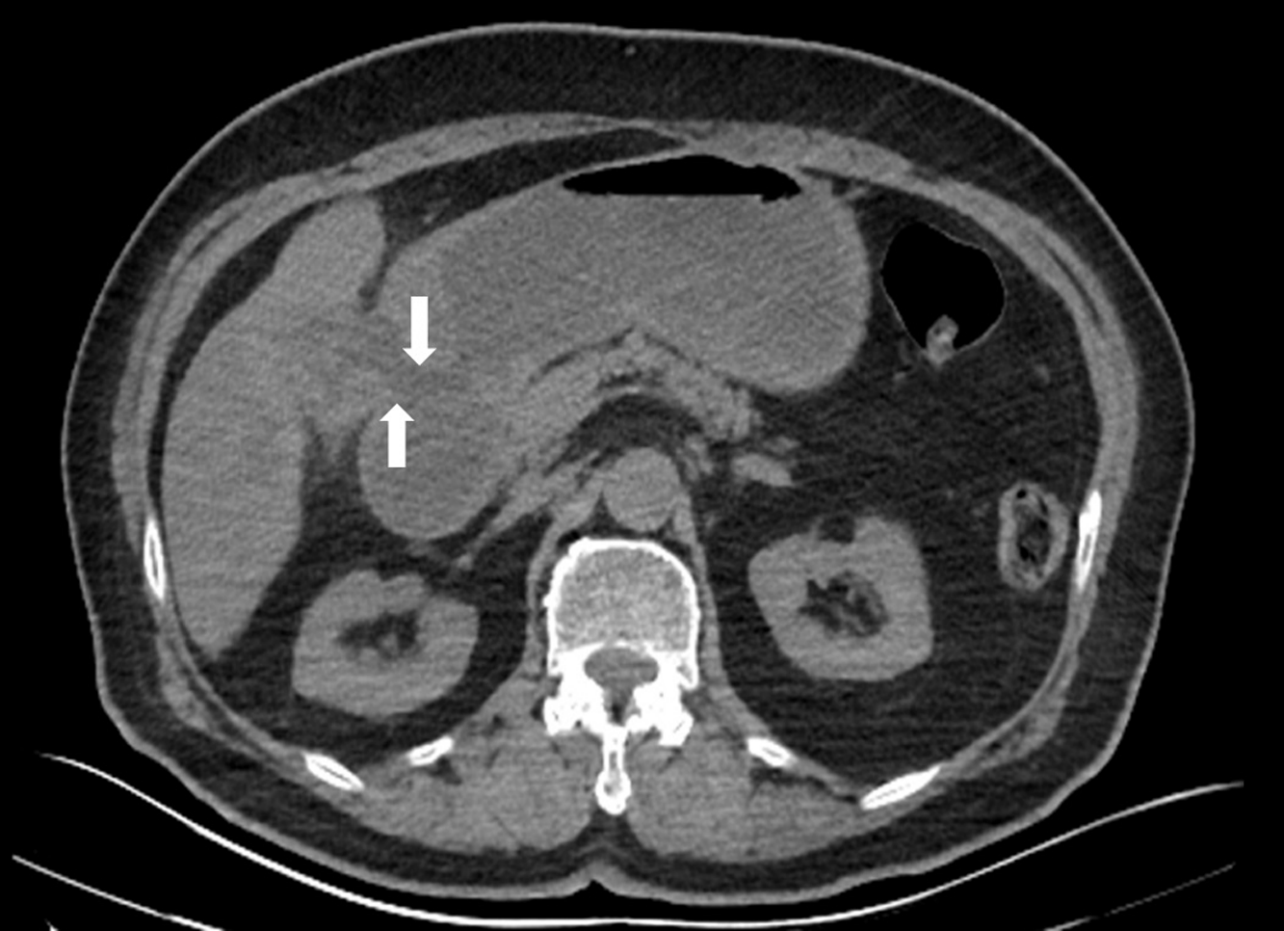
Cholecystoduodenal fistula tract (arrows).

**Figure 2.**
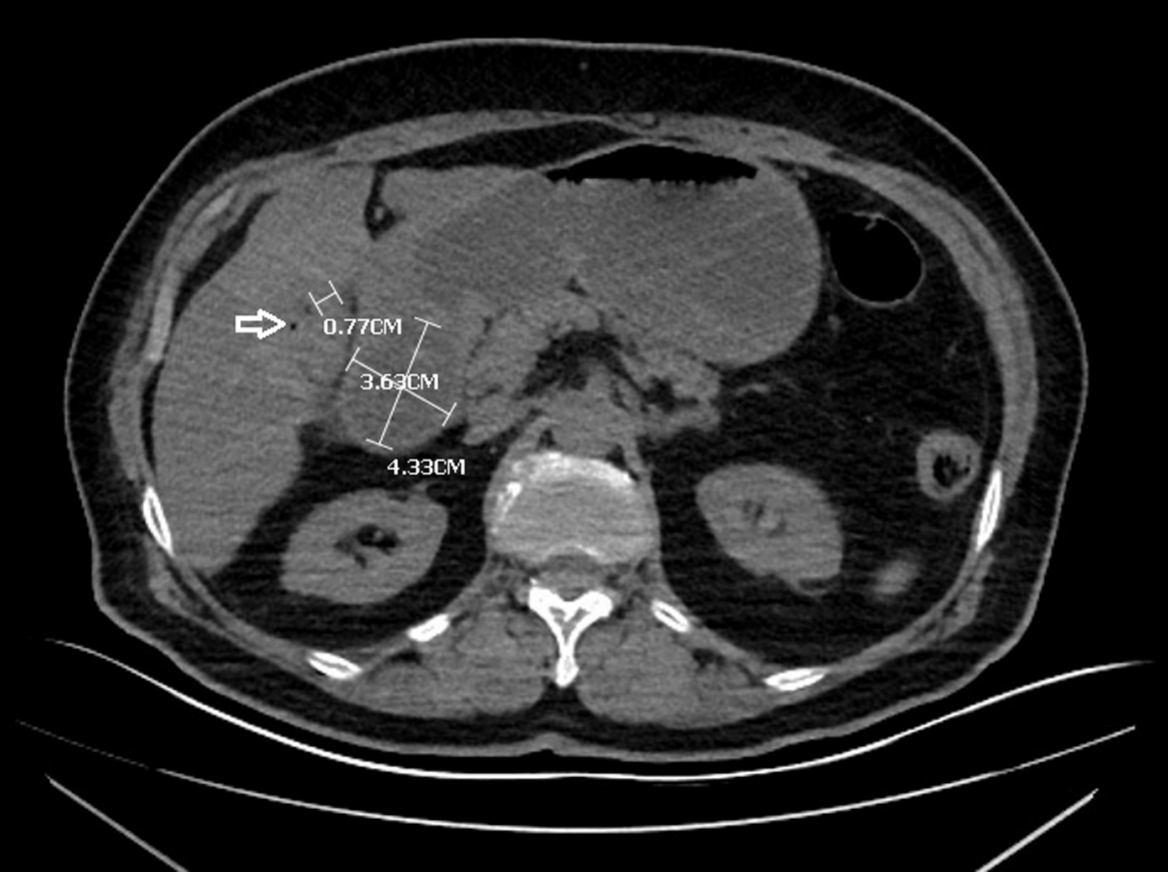
Free air in gallbladder (arrow), a noncalcified gallstone (4.33 cm × 3.63 cm) visualized within bulb of duodenum, dilated stomach, gallbladder wall thickening (0.77 cm).

**Figure 3.**
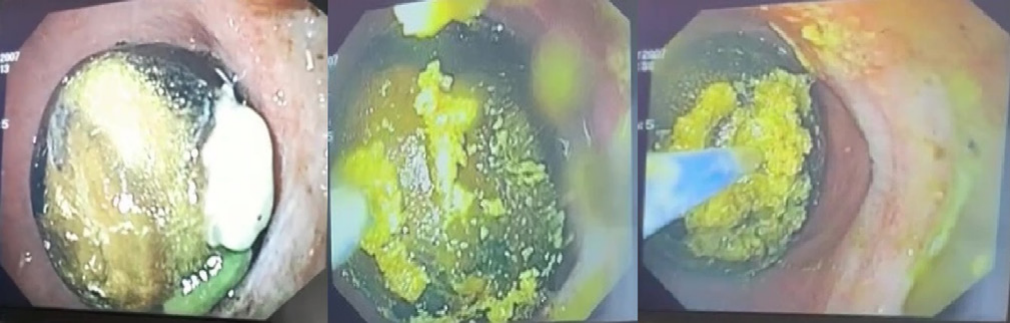
A giant gallstone in the bulb of duodenum and execution of laser lithotripsy.

**Figure 4.**
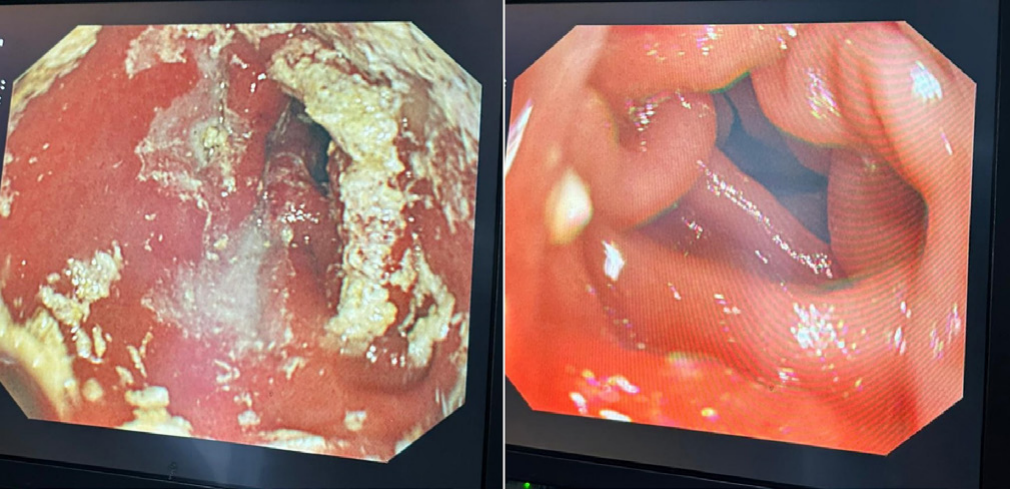
Duodenal passage provided after lithotripsy.

## References

[1] Haddad FG Mansour W Deeb L . Bouveret’s syndrome: literature review. Cureus. 2018;10(3):e2299. (10.7759/cureus.2299) 29755896 PMC5945273

[2] Caldwell KM Lee SJ Leggett PL Bajwa KS Mehta SS Shah SK . Bouveret syndrome: current management strategies. Clin Exp Gastroenterol. 2018;11:69 75. (10.2147/CEG.S132069) 29497323 PMC5819584

[3] Goonawardhana D Huynh R Rabindran J Becerril-Martinez G . Endoscopic lithotripsy for Bouveret syndrome complicated by small bowel obstruction secondary to gallstone fragments. J Surg Case Rep. 2021;2021(4):rjab118. (10.1093/jscr/rjab118) 33927858 PMC8055229

[4] Marks AJ Teichman JM . Lasers in clinical urology: state of the art and new horizons. World J Urol. 2007;25(3):227 233. (10.1007/s00345-007-0163-x) 17393172

